# High Efficiency Flat-Type GaN-Based Light-Emitting Diodes with Multiple Local Breakdown Conductive Channels

**DOI:** 10.3390/ma17112700

**Published:** 2024-06-03

**Authors:** Dae-Choul Choi, Seung Hun Lee, Sung-Nam Lee

**Affiliations:** Department of IT Semiconductor Convergence Engineering, Tech University of Korea, Siheung 15073, Republic of Korea

**Keywords:** GaN, LED, breakdown, efficiency

## Abstract

We investigated a flat-type p*-p LED composed of a p*-electrode with a local breakdown conductive channel (LBCC) formed in the p-type electrode region by applying reverse bias. By locally connecting the p*-electrode to the n-type layer via an LBCC, a flat-type LED structure is applied that can replace the n-type electrode without a mesa-etching process. Flat-type p*-p LEDs, devoid of the mesa process, demonstrate outstanding characteristics, boasting comparable light output power to conventional mesa-type n-p LEDs at the same injection current. However, they incur higher operating voltages, attributed to the smaller size of the p* region used as the n-type electrode compared to conventional n-p LEDs. Therefore, despite having comparable external quantum efficiency stemming from similar light output, flat-type p*-p LEDs exhibit diminished wall-plug efficiency (WPE) and voltage efficiency (VE) owing to elevated operating voltages. To address this, our study aimed to mitigate the series resistance of flat-type p*-p LEDs by augmenting the number of LBCCs to enhance the contact area, thereby reducing overall resistance. This structure holds promise for elevating WPE and VE by aligning the operating voltage more closely with that of mesa-type n-p LEDs. Consequently, rectifying the issue of high operating voltages in planar p*-p LEDs enables the creation of efficient LEDs devoid of crystal defects resulting from mesa-etching processes.

## 1. Introduction

III-nitride semiconductors have attracted great attention as a variety optical and electrical device due to their wide bandgap spanning the ultraviolet and visible-light spectrums and excellent physical and chemical properties [1,2]. They have been recognized for their potential in various applications such as telecommunications, solid-state lighting, and next-generation display sources [3,4,5]. Among these various applications, light-emitting diodes (LEDs) have emerged as one of the most extensively developed technologies and find ubiquitous applications across various aspects of daily life [6,7,8]. Therefore, the focus of research efforts to date has been on improving optical and electrical properties to achieve higher levels of optical output power and reliability [9,10,11]. Critical factors contributing to these efforts include junction temperature, heat dissipation, surface recombination, and reverse leakage current [12,13,14,15]. Notably, the properties of reverse leakage and breakdown are indispensable in ensuring the lifetime and reliability of LEDs, with a particular emphasis on mitigating electrostatic discharge damage [16,17]. Consequently, numerous researchers have focused their efforts on improving these parameters to enhance the long-term reliability of LEDs [9,10,11,12,13,14,15,16,17].

In terms of the breakdown phenomenon, an important issue in conventional LED reliability, failure of LEDs typically results in complete destruction, leading to the loss of typical diode I-V properties [18,19]. In our previous work, we suggested a local breakdown technique that induces partial rather than full breakdown in LEDs [20,21,22]. This localized breakdown phenomenon is caused by defect-assisted Zener breakdown in a very small region when a high reverse voltage is applied to areas with leakage currents, such as dislocations and surface V-defects [20]. In this region, conductive metals, such as indium in the InGaN active layer and metals in the transparent electrode, form channels connecting the p-type and n-type layers through evaporation and redeposition [21]. The p-electrode connects to the n-type GaN layer through the locally formed conductive channel. When a negative bias is applied to the p*-electrode, the local breakdown conductive channel (LBCC) behaves as if it were an n-type electrode. This innovative approach facilitated the formation of LBCCs, which served as efficient carrier injection paths [20]. Using the LBCC phenomenon, LEDs consisting of only p-type electrodes without n-type electrode where the p-type electrode (p*-electrode) with local breakdown region acts as electron supply path in place of n-type electrodes in GaN-based LEDs, contributing to the formation of localized breakdown conductive channels. The novel p*-electrode configuration, incorporating LBCCs, obviates the necessity for conventional n-type electrodes, thereby simplifying the manufacturing process of flat-type p*-p LEDs from mesa-structured n-p LEDs. This innovation can simplify the manufacturing process of flat-type p*-p LEDs by removing the need for n-type electrode, as seen in conventional mesa-structured n-p LEDs [21,22]. Despite these advantages, the very small size of the LBCCs in flat-type p*-p LEDs leads to higher resistance compared to conventional mesa-shaped n-p LEDs, which in turn increases the operating voltage and potentially reduces efficiency [22]. To address this issue, recent research has explored scaling down LED sizes to micro-LED dimensions, aiming to align the operating voltage of flat-type p*-p LEDs with that of mesa-type n-p LEDs, thereby enhancing the wall plug efficiency (WPE) [22]. However, for conventional LEDs larger than micro-LEDs, flat-type p*-p LEDs continue to exhibit high operating voltages. Therefore, it is proposed to increase the number of LBCCs to alleviate this problem. This study aims to enhance the optical and electrical properties of p*-p LEDs by incrementally increasing the number of LBCCs. By optimizing the design and configuration of flat-type p*-p LEDs to demonstrate improved performance, we propose the potential for replacing the conventional mesa-type n-p LEDs with a more efficient flat-type p*-p LED structure.

## 2. Materials and Methods

**Figure 1:**
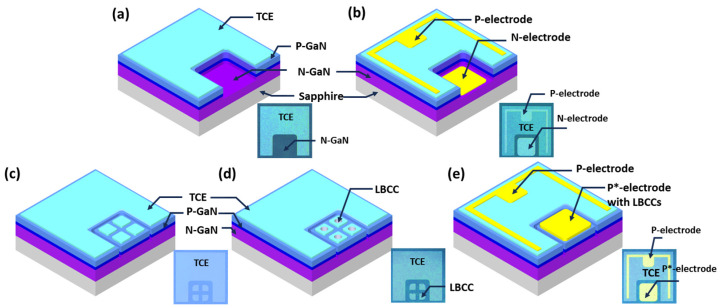
A schematic diagram of the fabrication method of the mesa-structure n-p LED (**a**,**b**). A schematic diagram of the fabrication method of the flat-type p*-p LED (**c**–**e**). The inset images at the bottom right are optical microscopy images of the LEDs for each fabrication process.

A 2.0 µm thick (0001) GaN template was grown on a c-plane sapphire substrate using a conventional two-step growth method employing metalorganic chemical vapor deposition. The growth process utilized trimethylgallium, trimethylindium, and ammonia as sources for Ga, In, and N, respectively. Silane and biscyclopentadienylmagnesium were used as n- and p-type dopants, respectively. Subsequent to the growth of the 2.0 µm thick GaN template, a standard n-p heterojunction LED structure was grown, comprising a 3.0 µm thick Si-doped n-type GaN layer, five-period In_0.15_Ga_0.85_N/GaN quantum wells, and a 0.1 µm thick Mg-doped p-type GaN layer. Following the growth of LED wafer, LED chips were fabricated, including conventional mesa-type n-p LEDs with lateral electrodes [22]. For the LED structures, Ti/Al and Ni/Au were utilized as n-type and p-type electrodes, respectively, deposited by an e-beam evaporator. Figure 1a,b shows the fabrication steps of a conventional n-p LED, while Figure 1c–e illustrates the fabrication process of a flat-type p*-p LED with a p*-layer with four LBCCs formed on the p-electrode. Figure 1a shows a transparent conductive electrode (TCE) deposited on top of a p-GaN layer, which is then mesa-etched to form the n-type electrode. The n-type electrode (Ti/Al) is then deposited on the mesa-etched area, as shown in Figure 1b. Compared to conventional mesa-structured n-p LEDs, a flat-type p*-p LED does not undergo the mesa-etching process to form n-type electrodes, as depicted in Figure 1c–e. The omission of the etching process, which can form these large areas of n-type electrodes, leaves a layer of p-GaN that can form additional LBCCs. To make multiple LBCCs, individual small p-type electrode regions were formed into one, two, three, and four LBCCs, respectively, and Au, a bonding metal, was deposited on top to them connect the small p*-electrodes that formed the LBCCs so that they could be used as one large p*-electrode. Figure 1c is a schematic showing the deposition of TCE directly onto a p-GaN layer without the mesa-etching process used in the fabrication of the n-p LED. As shown Figure 1d, the process formed four 60 µm sized p*-electrodes, allowing up to four LBCCs to be formed on a single LED. Finally, Figure 1e is a schematic of the final flat-type p*-p LED, showing the simultaneous deposition of Ni/Au bonding metal on the p*-electrodes with LBCCs and TCE layer on the p-layer. With this approach, a p*-p LED with multiple BCCs can be manufactured by additionally depositing p-type electrodes in a configuration encompassing all 60 × 60 µm^2^ divided electrodes. Consequently, the divided electrodes are interconnected in parallel due to the inclusion of an additional 120 × 120 µm^2^ sized electrode deposition.

For electrical characterization, voltage and current measurement of the LEDs were conducted using a Keithley 2400 source meter to explore the local breakdown phenomenon within the p-layer of both the n-p and p*-p LEDs. In addition, the L–I–V measurements were performed using an HP-4155 parameter analyzer (Agilent Technologies, Santa Clara, CA, USA). Light-emitting imagery was captured utilizing a Sony NEX-5 camera. In the efficiency analysis of all LEDs, external quantum efficiency (EQE), wall plug efficiency (WPE), and voltage efficiency (VE) were mainly analyzed. The pulse current was injected to compare the difference in light output power between the continuous wave (CW) and pulse driving for each LED using a precision pulsed current source (LDP-3811, Newport Corporation, Irvine, CA, USA). A Hot Chuck Controller (MST-1000H, MSTECH Corporation, Hwaseong, Republic of Korea) was employed to ascertain the characteristic temperature and junction temperature of the p*-p LEDs, with a focus on the influence of the number of LBCCs. The temperature varied systematically from 20 °C to 100 °C. Concurrently, the decreasing activation energy of light intensity was investigated as a function of temperature. This experimental approach facilitated a comprehensive understanding of the thermal behavior and performance dynamics of the p*-p LEDs, particularly in relation to the variation in LBCCs and temperature.

## 3. Results and Discussion

**Figure 2:**
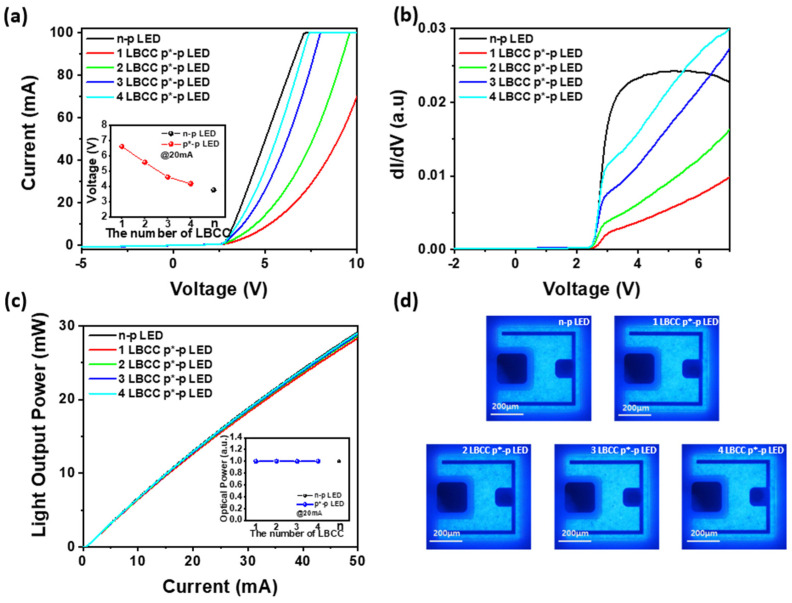
(**a**) Current (I)–voltage (V) curves, (**b**) instantaneous conductance (dI/dV)–voltage curves, (**c**) light output power (L)–current (I) curves, (**d**) light emission images at 2.0 mA of an n-p LED and 1- to 4-LBCC p*-p LED. Inset of (**a**) operating voltages n-p LED and 1- to 4-LBCC p*-p LED at 20 mA.

In the flat-type p*-p LED structure, the anode and cathode currents were directed to the p-layer and p-layer, respectively, as shown in Figure 1c, leading to the formation of an LBCC region within the p-layer. Initially, applying a low injection current of approximately 0.3 mA resulted in a high operating voltage reaching up to −50 V, reminiscent of the behavior observed in conventional n-p LEDs under reverse bias conditions. However, as the operating current surpassed 1.0 mA, the operating voltage of the p*-p LED suddenly decreased from over 50 V to a few volts, and the operating voltage remained a few volts even after the operating current decreased below 1.0 mA [20]. This phenomenon indicates that there is a distinct change in the electrical behavior of the LED structure due to the formation of LBCCs within the p-region, resulting in the formation of a p*-layer. The p*-electrode is interconnected with the n-type layer, effectively functioning as an alternative to a conventional n-type electrode without necessitating a mesa-etching process [20,21,22]. In this process, as only one LBCC can be generated from a single LED, we incorporated one to four additional p-electrodes to establish multiple LBCCs, as illustrated in Figure 1c,d. Subsequently, to interconnect these individual LBCCs into a unified electrode, a top electrode was deposited, facilitating the parallel connection of each LBCC, as shown in Figure 1e. This method is expected to improve the efficiency of the LED by reducing the series resistance by paralleling high-resistance LBCCs within a single LED. Figure 2a shows the current (I)-voltage (V) curves of an n-p LED and p*-p LED featuring different numbers of LBCCs, from one to four. Notably, regardless of the number of LBCCs, p*-p LEDs exhibit a distinct turn-on voltage of approximately 2.6 V, about the same as conventional n-p LEDs, indicating that the LBCCs in the p*-p LED behave similar to the n-electrodes of the n-p LEDs [20]. Since the LBCC formed on the p*-electrode is only responsible for injecting n-type carriers into the n-type layer, n-p LEDs and p*-p LEDs have the same diffusion voltage, which can explain why both types of LEDs exhibit the same turn-on voltage. However, the operating voltage, measured at an applied current of 20 mA, varies significantly between the n-p LED and the p*-p LED with one LBCC. In particular, the operation voltage of an n-p LED is approximately 3.75 V, whereas for the p*-p LED with one LBCC, it is around 6.6 V. This substantial disparity can be attributed to the narrower pathway provided by the LBCC compared to the n-type electrode in the n-p LED. Essentially, the mismatch in contact area between the p*-electrode, from which LBCCs with diameters of less than 10 μm are formed, and the n-electrode, which is tens of μm in size, is responsible for this significant operating voltage difference [21]. However, as the number of LBCCs increases, the disparity in operating voltage decreases, as shown in the inset of Figure 2a, indicating that this gap is likely to be mitigated as the number of LBCCs increases. The operating voltages of the p*-p LEDs with varying numbers of LBCC are as follows: two-LBCC p*-p LED: 5.56 V, three-LBCC p*-p LED: 4.6 V, and four-LBCC p*-p LED: 4.16 V. This trend occurs because, as the number of LBCCs connected in parallel increases, the path for the n-type carriers is expanded, which relaxes the resistance difference between the n-electrode and the p*-electrode. Assuming that the operating voltage of the n-p LED is 100%, the operating voltage of the one-LBCC p*-p LED is 176%, which is a significant increase compared to the n-p LED. However, when the number of LBCCs is increased to four, the operating voltage decreases to 110.9%, which is a significant improvement over the one-LBCC p*-p LED. This suggests that by increasing the number of LBCCs in a p*-p LEDs from one to four, the operating voltage can approach similar levels to that of the n-p LED, as shown in the inset of Figure 2a. Figure 2b illustrates the derivative of voltage with respect to current, which shows the ratio of instantaneous conductance (dS = dI/dV) at each voltage increase. This provides insight into the dynamic changes in conductivity as voltage increases across the LED. The behavior observed in the n-p LEDs, with a rapid increase in instantaneous conductance at low voltages followed by a near-constant value after 3 V, is indicative of the characteristic response of a typical LED. Initially, at low voltages, there is a sharp rise in conductance as the LED begins to conduct current. Once the LED reaches a certain voltage threshold (around 3 V in this case), it enters the forward-bias region, where the increase in voltage does not significantly affect the conductance, resulting in a relatively constant value. In contrast, the p*-p LED exhibits a different behavior, with an initial very low increase in instantaneous conductance followed by a more gradual increase with a relatively constant slope. This behavior suggests that the p*-p LED operates differently from a traditional n-p LED. The observed trend in the instantaneous conductance of the p*-p LED, initially low but increasing with voltage, suggests a decrease in resistance as voltage rises, likely attributed to joule heating effects within the device. As the voltage increases, the temperature of the LED increases, leading to a reduction in resistance and consequently an increase in conductance [23]. Moreover, as the number of LBCCs in the p*-p LED increases, the instantaneous conductance exhibits a steady rise at a constant voltage. This phenomenon can be attributed to the augmented pathway provided by the increased number of LBCCs for the flow of n-type carriers. With more LBCCs, there is a greater availability of pathways for electron movement, resulting in a decrease in resistance and subsequently an increase in conductance at a given voltage level. Figure 2c shows the light output power of a conventional n-p LED and p*-p LEDs with different numbers of LBCCs. The light output powers of p*-p LEDs are very similar to that of an n-p LED, regardless of the number of LBCCs [20,21]. This similarity stems from utilizing the same LED wafer for both types of LEDs, featuring an identical InGaN active layer and n/p-type layers. However, a crucial distinction lies in the injection of n-type carriers: in n-p LED, they are injected through the n-type electrode, whereas in the p*-p LEDs, this function is performed by the LBCCs. Despite this difference, the shared active layer and p-type layer ensure consistent light emission between the two LED types. LBCCs effectively function as n-type electrodes, providing a sufficient pathway for n-type carrier injection into the active region of LEDs. At injection currents exceeding 20 mA, the light output of p*-p LEDs is marginally lower than that of n-p LEDs. However, there is a slight increase in light output power with an increase in the number of LBCCs. Remarkably, a p*-p LED with four LBCCs displays nearly identical light output power to an n-p LED. This phenomenon is attributed to the degradation of optical intensity caused by heat generation resulting from high resistance, as LBCCs offer limited area for electron transport paths. Despite experiencing some light intensity degradation at high currents, p*-p LEDs demonstrate light output characteristics that closely resemble those of n-p LEDs. This underscores the effectiveness of LBCCs as a viable alternative injection route.

In Figure 2d, light emission images of both an n-p LED and p*-p LED, controlled by varying numbers of LBCCs, are displayed under an applied current of 2.0 mA. It is evident that n-p LEDs and p*-p LEDs with different LBCCs exhibit almost similar electroluminescence behaviors. This finding implies that the light emission intensity of the p*-p LED remains consistent regardless of the number of LBCCs, mirroring the characteristics observed in Figure 2c. Consequently, despite varying numbers of LBCCs, the p*-p LED exhibits light output characteristics similar to those of the n-p LED, highlighting the viability of LBCCs as alternative injection pathways.

**Figure 3:**
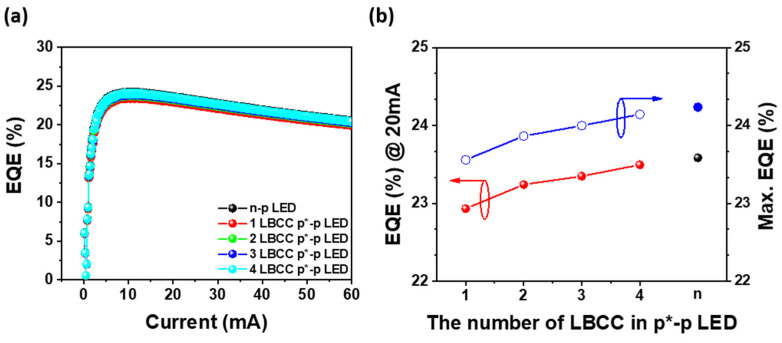
(**a**) EQE of n-p LED and p*-p LEDs as a function of injection current, (**b**) EQE at 20 mA and maximum EQEs of n-p LED and p*-p LEDs with different LBCCs.

The external quantum efficiency (EQE) of the n-p LED and one- to four-LBCC p*-p LED, plotted as a function of the injected current, is depicted in Figure 3a. It is evident that all LEDs exhibited a similar maximum EQE of approximately 24% at an injection current of 10 mA. This consistency arises from the fact that, as demonstrated in Figure 2c, each LED displayed similar light output power irrespective of the number of LBCCs or the electrode type (n or p*). EQE, defined as the number of photons emitted into free space per unit of time for a given number of injected electrons [24], yields nearly identical values for the n-p LED and 1–4-LBCC p*-p LEDs sharing the same emitters at equivalent currents. However, as illustrated in Figure 2a, the EQE of a one-LBCC p*-p LED with the highest operating voltage of p*-p LEDs is about 0.7% lower than that of an n-p LED. This is similar to the slight decrease in light output power observed in p*-p LEDs at a higher injection current region, which is attributed to diminished optical efficiency due to resistive heating in a p*-p LED with one LBCC [25,26,27,28]. In addition, the current required to achieve maximum EQE is found to be constant at approximately 10.5 mA for both n-p LEDs and p*-p LEDs, irrespective of the number of LBCCs. In Figure 3b, the EQE at an injection current of 20 mA follows a similar trend to the maximum EQE, with EQE values increasing as the number of LBCCs increases, culminating in a p*-p LED with four LBCCs exhibiting a similar value to the n-p LED. Furthermore, both the n-p LED and p*-p LEDs with one to four LBCCs exhibit an EQE droop of approximately 12.5%, regardless of the number of LBCCs or LED type. This is because the EQE is dependent on the injected current and light output power, irrespective of the operating voltage.

**Figure 4:**
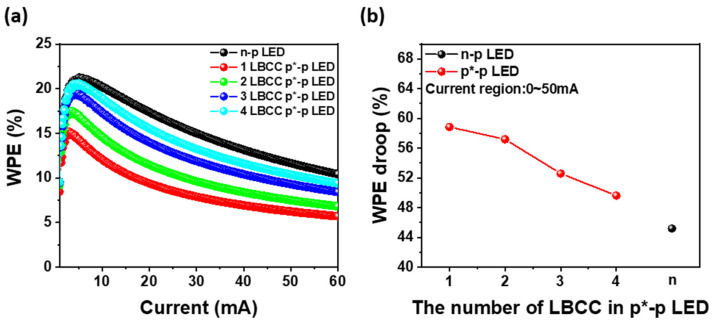
(**a**) WPE of n-p LED and 1 to 4 LBCC p*-p LED as a function of injection current and (**b**) WPE droop of both type LEDs obtaining from 0 to 50 mA.

The p*-p LEDs, which are notably influenced by the operating voltage, were additionally analyzed to determine the efficiency of both types of LEDs concerning the operating voltage. The wall plug efficiency (WPE) is the efficiency with which an LED converts electrical power into light [24], measured as a function of power, including operating voltage and current. In Figure 4a, the WPEs of the n-p LED and one- to four-LBCC p*-p LEDs are plotted against injection current. It illustrates the maximum WPE for each LED occurring at approximately 5.0 mA. Notably, the maximum WPE for n-p LED reaches 21.3% at 5.12 mA. However, the one-LBCC p*-p LED exhibits maximum WPE of 15.2% at 2.72 mA, which is inferior to its EQE counterpart. It can be observed that the operating voltage significantly influences the WPE, particularly evident in one-LBCC p*-p LEDs, which demonstrate a lower WPE due to their higher operating voltage compared to n-p LEDs. However, as shown in Figure 2a, in the case of a p*-p LED utilizing only one LBCC, the narrower pathway for n-type carriers relative to the n-p LED leads to higher operating voltages and lower WPE [22]. However, a four-LBCC p*-p LED achieves a maximum WPE of 20.6% at 5.12 mA, which is very similar to the maximum WPE of the n-p LED. As the number of LBCCs increases, the pathway for an n-type carrier within the p* electrode widens, reducing the operating voltage of the p*-p LED to levels akin to the n-p LED, resulting in comparable WPE between the two LED types. As shown in Figure 4a, at an injection current of 20 mA, the WPE of the n-p LED is 17.3%, which surpasses that of the p*-p LED. However, as the LBCC increases from one to four, the WPE of the p*-p LED increases from 9.4% to 15.5%, reducing the difference with the WPE of the n-p LED. Furthermore, the maximum WPE of n-p LEDs is 21.3%, which is higher than that of p*-p LEDs, but as LBCC increases from one to four, the maximum WPE of p*-p LEDs increases from 15.2% to 20.6%, which significantly reduces the difference with the WPE of n-p LED. Additionally, owing to the limited LBCC of the p*-electrode, a one-LBCC p*-p LED exhibits a significant WPE droop (WPE_max_-WPE_50mA_) of approximately 58.9%, as shown in Figure 4b [22,29]. Nonetheless, as the number of LBCCs increases to four, the WPE droop is reduced to about 49.5% because the carrier path area is enlarged, and the series resistance is reduced. This indicates that the gap between n-p LEDs, characterized by a WPE droop of approximately 45.2%, and p*-p LEDs, displaying a WPE droop of 49.5%, has narrowed to just 4%, rendering them remarkably similar. This significant reduction in difference from the WPE of n-p LEDs by increasing the number of LBCCs in p*-p LEDs indicates that there is fewer resistance issue when supplying n-type carriers, potentially allowing the WPEs of p*-p LEDs to match that of an n-p LED using a conventional n-electrode.

**Figure 5:**
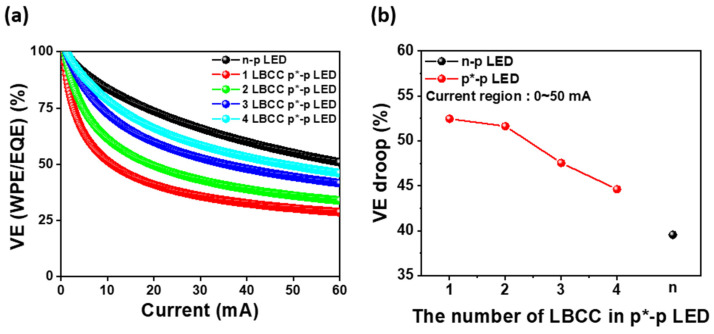
(**a**) VE of n-p LED and 1- to 4-LBCC p*-p LEDs as a function of injection current and (**b**) VE droop of both type LEDs obtaining from 0 to 50 mA.

Figure 5a depicts the voltage efficiency (VE) of the n-p LED and one- to four-LBCC p*-p LEDs, which is calculated from the ratio of WPE to EQE and is affected by the operating voltage [22]. At the operating current of 5.0 mA, the n-p LED exhibits a VE of 90.9%, whereas the one-LBCC p*-p LED demonstrates a relatively low VE of 63.4%. However, the two-LBCC p*-p LED achieves a VE of 74.3%, the three-LBCC p*-p LED attains 84.5%, and the four-LBCC p*-p LED reaches 88.7%, approaching the performance level of the n-p LED as the number of LBCCs increases. This trend is attributed to the fact that, as the LBCC increases, the path of the n-type carrier expands, which reduces the difference in contact area between the n-type electrode and the p*-electrode, as shown in Figure 2a, resulting in a decrease in the operation voltage. Moreover, at injection currents above 20 mA, the VE exhibits a similar pattern to that at 5.0 mA. Figure 5b is a graph of VE droop against the number of LBCCs, demonstrating that VE droop decreases as the number of BCCs increases. This trend closely mirrors the observations for WPE, highlighting the consistent improvement in performance at high injection currents as the number of LBCCs in the p*-p LED increases. These results underscore that WPE and VE characteristics can be improved to a level comparable to an n-p LED by extending the path of n-type carriers to reduce resistance.

**Figure 6:**
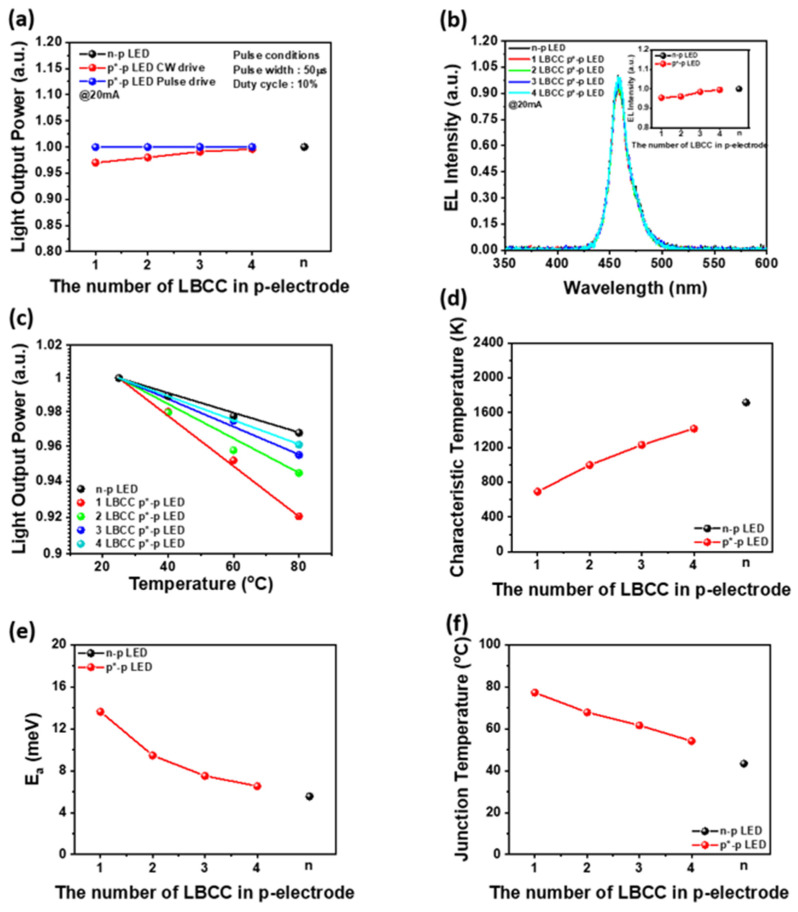
(**a**) Light output powers of n-p LED and p*-p LED with 1 to 4 LBCCs under CW and pulse operation. (**b**) EL spectrum of n-p LED and p*-p LED with 1 to 4 LBCCs at 20 mA. (**c**) Normalized output power of n-p LED and p*-p LEDs with 1 to 4 LBCCs as a function of the ambient temperatures. (**d**) Characteristic temperatures (T_1_), (**e**) activation energy, and (**f**) junction temperature of n-p LED and p*-p LEDs with 1 to 4 LBCCs.

As evident from Figure 3, Figure 4 and Figure 5, the EQE droop remains relatively constant regardless of the number of LBCCs, while both WPE and VE droop decrease as the number of LBCCs increases in p*-p LEDs, converging to similar values for n-p LEDs. This phenomenon is attributed to the LBCCs having a smaller carrier path area, a characteristic that contributes to lower efficiency due to resistive heating caused by carrier migration. To validate the resistive heating characteristic of p*-p LEDs with different numbers of LBCCs, the change in emission intensity with pulse driving and ambient temperature variation is observed, as depicted in Figure 6a,b. Notably, Figure 6a presents a comparison of the difference in light output power between continuous wave (CW) and pulse driving for each LED. It illustrates that the light output power decreases with a smaller number of LBCCs, which can be attributed to the poor thermal performance due to the high series resistance of the p*-electrode with a very small LBCC [30,31]. To validate this phenomenon, measurements were performed under pulse condition of 50 µs pulse width and 10% duty cycle, which are less affected by the thermal heating process. The comparison confirms that when driven by pulse, the light output power of both p*-p LED and the n-p LED becomes more similar compared to the CW-driven process. Figure 6b presents an electroluminescence (EL) spectrum for each LED at 20 mA. It illustrates that EL intensity of a p*-p LED decreases with a lower number of LBCCs. This can also be attributed to the poor thermal performance due to the high series resistance of the p*-electrodes with a very small LBCC. Figure 6c illustrates the normalized decrease in emission intensity with increasing the ambient temperature, irrespective of LED types. However, the emission intensity of the p*-p LED exhibits a more pronounced decrease with increasing ambient temperature compared to the n-p LED. Furthermore, the decrease ratio of emission intensity in the p*-p LED diminishes with an increase in the number of LBCCs. In general, the reduction in light emission intensity with increasing temperature can be attributed to various temperature-dependent factors, including non-radiative recombination through deep levels, surface recombination, and carrier loss to heterostructure barriers [10,14,32]. However, as both n-p LEDs and p*-p LEDs are fabricated on the same wafer and share the same crystal defects and thin film structure, it is believed that the decrease in luminous efficiency is solely attributable to heat generation resulting from the increase in operating voltage with the introduction of LBCCs. Therefore, increasing the number of LBCCs in p*-p LEDs is believed to mitigate the temperature-related reduction of emission efficiency due to the resistive heating process generated by the small LBCC region. In addition, the temperature-dependence of the emission intensity near room temperature can be described by the characteristic temperatures of an n-p LED and a p*-p LED using the following phenomenological equation [24].

(1) $$I=I_{300K}(\exp-\frac{\left(T-300K\right)}{T_{1}})$$

where I and I_300K_ are the emission intensity at temperature T and 300 K, respectively, and T_1_ is a characteristic temperature. The characteristic temperatures of the n-p LED and one- to four-LBCC p*-p LED are shown in Figure 6d. The characteristic temperature of the one-LBCC p*-p LED is 690 K, while that of the two-LBCC p*-p LED is 997 K, the three-LBCC p*-p LED is 1229 K, and the four-LBCC p*-p LED is 1415 K. The lower the number of LBCCs, the lower the characteristic temperatures, indicating a higher temperature dependence. This trend is attributed to the heat generated by the high series resistance of the LBCC in the p*-electrode, which contributes to the increase in temperature and subsequently reduces emission intensity [33]. The high series resistance associated with the small area of the LBCC in the p*-electrode increases the heating process of the LED itself, resulting in more non-radiative recombination, which enhances the emission intensity reduction and makes it more temperature-dependent [34,35]. In particular, the characteristic temperature of the one-LBCC p*-p LED shows a very low value of 40.3% compared to the n-p LED, while the characteristic temperature of the four-LBCC p*-p LED increases to 82.5% compared to the n-p LED. It means that multiple LBCCs are believed to be effective in improving the emission efficiency by decreasing the resistive heating problem in the p*-p LED. Figure 6e illustrates the activation energies of an n-p LED and one- to four-LBCC p*-p LEDs obtained from Figure 6c using the Arrhenius Equation (2):

(2) $$lnP\propto -E_{a}/(k_{B}T)$$

where E_a_ represents the activation energy, P is the optical output power, k_B_ is Boltzmann’s constant, and T is the absolute temperature. The n-p LED exhibits an activation energy of 5.55 meV, while the one-LBCC p*-p LED, two-LBCC p*-p LED, three-LBCC p*-p LED, and four-LBCC p*-p LED show activation energies of 13.62 meV, 9.46 meV, 7.51 meV, and 6.53 meV, respectively. As the number of LBCCs in the p*-p LED increases, the activation energy decreases and approaches that of the n-p LED. Activation energy typically refers to the minimum energy needed to transition from one state to another. In a p*-p LED with a single LBCC, the activation energy of 13.6 meV is more than twice the value of 5.55 meV, which represents the activation energy for temperature-dependent emission reduction at the n-type electrode in an n-p LED. However, as illustrated in Figure 2a, it is believed that as the LBCC increases, the series resistance of the p*-electrode decreases, which reduces the potential barrier for carrier transport and thereby enhances current flow. Therefore, the formation of multiple LBCCs indicates that the joule heating generated in the n-type carrier supply is reduced, which can be improved to an optical efficiency comparable to that of conventional n-p LEDs. Figure 6f illustrates the junction temperatures between the n-p LED and one- to four-LBCC p*-p LEDs at the operation current of 20 mA. The junction temperatures are measured by a forward voltage method, i.e., pulse-calibration measurement (a pulse width of 10 µs and a duty cycle of 1.0%) with operating temperature varied from 20 °C to 100 °C and determination of junction temperature measurement using different DC currents [22]. The junction temperature of the one-LBCC p*-p LED is 77.3 °C, while the junction temperature of the four-LBCC p*-p LED is 54.2 °C. Both are higher than the junction temperature of the n-p LED, which is 43.4 °C. When the number of LBCCs is one, the area of the LBCC is narrow, indicating a high junction temperature due to high vertical resistance and increased resistance heating. However, as the number of LBCCs increases, the area of the LBCCs becomes larger, facilitating carrier movement and reducing vertical resistance. This reduction in resistance decreases heat generation, resulting in a lower junction temperature. It is believed that this improvement in thermal characteristics results in the performance improvement of the p*-p LED using an LBCC.

## 4. Conclusions

We investigated the characteristics of a p*-p LED as the number of LBCCs, serving as a pathway for n-type carriers instead of n-electrodes, increased, comparing them with n-p LEDs. With the number of LBCCs rising from one to four, the operating voltage at 20 mA decreased from 6.6 V to 4.16 V, aligning it closely with that of the n-p LED. Although a slight decrease in light output power occurred due to thermal degradation from the high series resistance of the p*-electrode, the light output power of the four-LBCC p*-p LED remained largely consistent with that of n-p LEDs. Regardless of the number of LBCCs, p*-p LEDs exhibited similar efficiency to n-p LEDs in terms of EQE, which is dependent on light output power, and WPE, which is affected by operating voltage, with higher maximum WPE and lower WPE droop observed as the number of LBCCs increased. Similarly, VE, influenced by operating voltage, yielded similar results to WPE. As the number of BCCs increased from one to four, VE at 5.0 mA increased from 63.7% to 88.7%, and VE droop decreased from 52.5% to 44.6%. Additionally, the characteristic temperature increased with the number of LBCCs, indicating a temperature dependence of the optical intensity decreased due to a decrease in the junction temperature. Moreover, as the number of LBCCs increased, activation energy decreased, enhancing current flow, by addressing the chronic problem of LBCCs with high series resistance due to the small region of the p*-electrode, and the p*-p LED demonstrated performance comparable to that of the n-p LEDs.

## Data Availability

Data are contained within the article.
